# Neoadjuvant cabozantinib restores CD8+ T cells in patients with locally advanced non-metastatic clear cell renal cell carcinoma: a phase 2 trial

**DOI:** 10.21203/rs.3.rs-4849400/v1

**Published:** 2024-08-08

**Authors:** Mehmet A. Bilen, BaoHan T. Vo, Yuan Liu, Rachel Greenwald, Amir H. Davarpanah, Donald McGuire, Rakesh Shiradkar, Liping Li, Bassel Nazha, Jacqueline T. Brown, Sierra Williams, Wilena Session, Greta Russler, Sarah Caulfield, Shreyas S. Joshi, Vikram M. Narayan, Christopher P. Filson, Kenneth Ogan, Omer Kucuk, Bradley Curtis Carthon, Luke Del Balzo, Athena Cohen, Adriana Boyanton, Nataliya Prokhnevska, Maria Andrea Cardenas, Ewelina Sobierajska, Caroline S. Jansen, Dattatraya H. Patil, Edouard Nicaise, Adeboye O. Osunkoya, Haydn Kissick, Viraj A. Master

**Affiliations:** 1 Winship Cancer Institute, Emory University, Atlanta, GA, USA.; 2 Department of Hematology and Medical Oncology, Emory University School of Medicine, Atlanta, GA, USA.; 3 Department of Urology, Emory University School of Medicine, Atlanta, GA, USA.; 4 Department of Biostatistics and Bioinformatics, Rollins School of Public Health, Emory University, Atlanta, GA, USA.; 5 Department of Radiology and Imaging Sciences, Emory University School of Medicine, Atlanta, GA, USA.; 6 Department of Microbiology and Immunology, Emory University School of Medicine, Atlanta, GA, USA.; 7 Emory Vaccine Center, Emory University, Atlanta, GA, USA.; 8 Wallace H. Coulter Department of Biomedical Engineering, Georgia Institute of Technology and Emory University, Atlanta, GA, USA.; 9 Department of Pharmaceutical Services, Emory University School of Medicine, Atlanta, GA, USA.; 10 Department of Pathology, Emory University School of Medicine, Atlanta, GA, USA.

## Abstract

Cabozantinib is an oral multikinase inhibitor approved for treatment in metastatic renal cell carcinoma (RCC). We hypothesized that neoadjuvant cabozantinib could downstage localized tumors, facilitating partial nephrectomy, and facilitating surgery in patients with locally advanced tumors that would require significant adjacent organ resection. We, therefore, conducted a phase 2, single-arm trial of cabozantinib treatment for 12 weeks in 17 patients with locally advanced biopsy-proven non-metastatic clear cell RCC before surgical resection. Six patients (35%) experienced a partial response, and 11 patients (65%) had stable disease. We identified that plasma cell-free DNA (cfDNA), VEGF, c-MET, Gas6, and AXL were significantly increased while VEGFR2 decreased during cabozantinib treatments. There was a trend towards CD8+ T cells becoming activated in the blood, expressing the proliferation marker Ki67 and activation markers HLA-DR and CD38. Cabozantinib treatment depleted myeloid populations acutely. Importantly, immune niches made up of the stem-like CD8+ T cells and antigen presenting cells were increased in every patient. These data suggest that cabozantinib treatment was clinically active and safe in the neoadjuvant setting in patients with locally advanced non-metastatic clear cell RCC and activated the anti-tumor CD8+ T cell response. The trial is registered at ClinicalTrials.gov under registration no. NCT04022343.

## Introduction

Kidney cancer has among the most rapidly rising incidence rates globally, and is particularly prevalent among young patients, and in minorities^1–3^. In the United States, approximately 81,610 new cases of renal cell carcinoma (RCC) will be diagnosed in 2024^4^ Of those patients, 30% will develop metastatic RCC^5^. The initial treatment of locally advanced disease is partial or radical nephrectomy. While surgery cures many patients, unfortunately around 50% of patients recur within 5 years^6^. Due to this high recurrence rate, there has been a growing trend to intensify therapy to improve these patients’ outcomes. Most notable is recent data indicating adjuvant anti-PD1 given after surgery has a small benefit on overall survival^7^. Given the benefit of treatment intensification, there is now an interest in determining if neoadjuvant approaches may have additional benefits.

Neoadjuvant therapeutic strategies were originally designed to reduce tumor size to allow less invasive surgical approaches, like allowing resection of previously unresectable tumors. In addition, some patients may become eligible for partial nephrectomy, also known as nephron-sparing surgery, which results in significant functional benefits by preserving renal function, making it a preferred choice for patients with pre-existing renal issues and reducing the long-term risk of renal insufficiency compared to radical nephrectomy. While this reduced surgical burden was the original goal of these therapies, there has been a growing recognition that some therapies delivered in the neoadjuvant setting improve the long-term survival of patients. This is particularly true for immunotherapies that might have long term effects on tumor control by stimulating a long-lasting anti-tumor immune response allowing treatment effects that extend far beyond the treatment window^8^.

Cabozantinib is a multi-tyrosine kinase inhibitor (TKI) of MET, AXL, RET, and VEGFR2 which reduces tumor growth, metastasis, and angiogenesis and is approved for use in patients with advanced RCC^9–11^. Importantly, in several preclinical models, cabozantinib has been found to increase the immune response against the tumor, and in some cases the anti-tumor effects rely on the presence of the adaptive immune response^12–15^. Because of the direct anti-tumor effect that could allow less severe surgical approaches, in addition to the potential increase in anti-tumor immunity, this compound is an attractive candidate to test in a neoadjuvant setting. To investigate, we conducted a phase 2 study of neoadjuvant cabozantinib in patients with locally advanced non-metastatic clear cell RCC (ccRCC). Patients with clinical stage ≥ T3Nx or TanyN+ or deemed unresectable by the surgeon with biopsy-proven ccRCC were eligible for this study and received cabozantinib at a starting dose of 60 mg daily for 12 weeks. The primary outcome was the objective response rate per Response Evaluation Criteria in Solid Tumors (RECIST) v1.1 (complete, partial responses, and stable disease) at week 12 after the administration of cabozantinib as determined by independent radiologist review. Secondary outcomes included safety, tolerability, clinical and surgical outcomes, and quality of life. We also evaluate the correlative studies by determining the functional and phenotypic changes in T cells or myeloid cell markers in patient peripheral blood and tumors after treatment.

## Results

### Cabozantinib reduces tumor size and is safe in the neoadjuvant setting in ccRCC

**Extended Data Fig. 1|:**
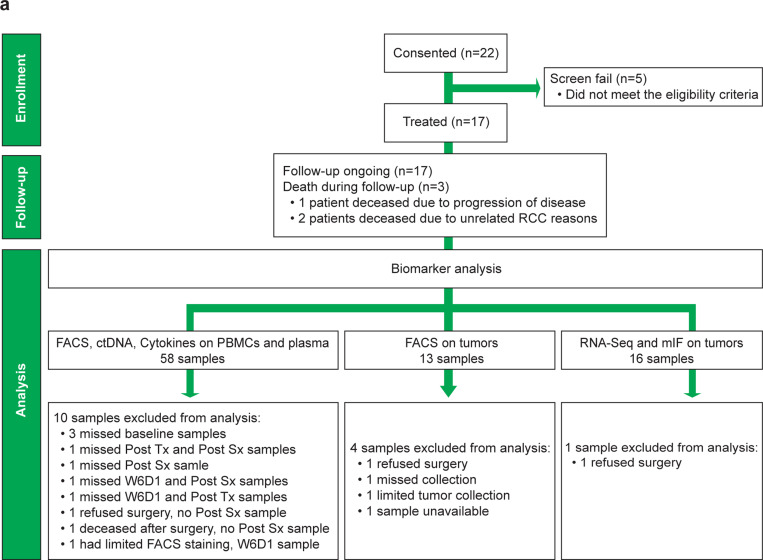
The study CONSORT diagram. **a**, Flow chart showing details of patients who participated in the study. CONSORT, Consolidated Standards of Reporting Trials.

**Fig. 1|:**
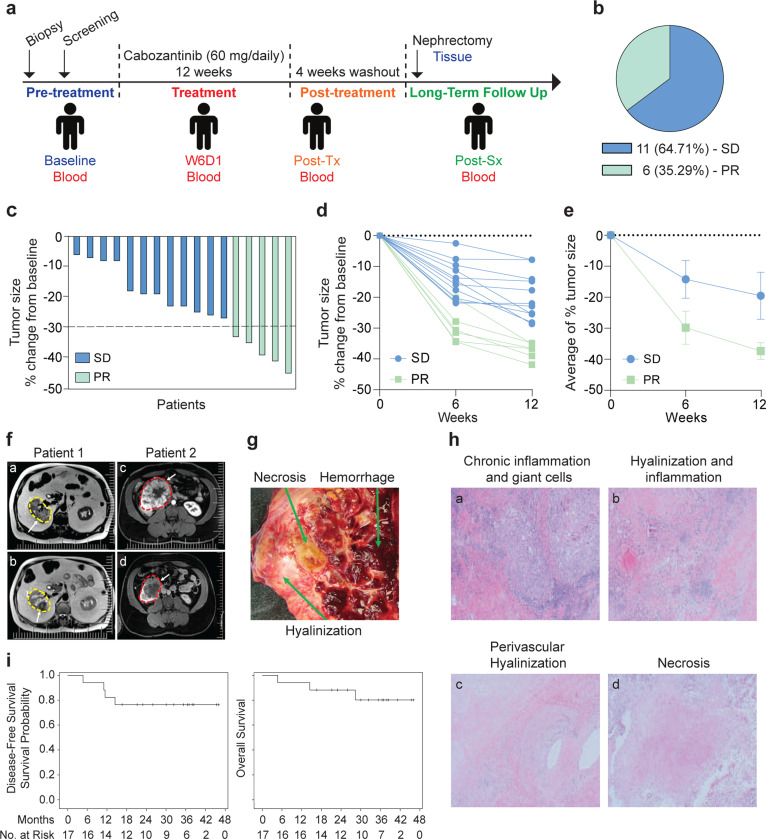
Clinical outcomes of ccRCC patients receiving cabozantinib treatment. **a**, Study design of patients with ccRCC were enrolled to receive neoadjuvant cabozantinib for 12 weeks before surgical resection. Peripheral blood was collected at baseline, W6D1, Post Tx, and Post Sx. **b**, Patients were assessed for clinical response using RECIST criteria. **c**, Waterfall diagram describing the percentage change in tumor size at week 12 of SD (n = 11) and PR (n = 6) patients. **d**, Spider plot showing the percentage change in tumor size at week 6 and 12 of SD and PR patients. **e**, Spider plot shows the average tumor size of a patient’s response to SD and PR. **f**, MRI imaging showing patient 1 was converted from radical to partial nephrectomy. Patient 2 was deemed to be unresectable became resectable at the end of treatment. **g**, Tumor sample showing area of necrosis, hemorrhage, and hyalinization. **h**, H&E staining of a) chronic inflammation and giant cells, b) hyalinization and inflammation, c) perivascular hyalinization and d) necrosis. **i**, DFS and OS for the 17 treated patients. One-year DFS was 82.4% (95% CI = 54.7% - 93.9%). One-year OS was 94.1% (95% CI = 65.0% - 99.1%). ccRCC, clear cell renal cell carcinoma; W6D1, week 6 day 1; RECIST, Response Evaluation Criteria in Solid Tumor; PR, partial response; SD, stable disease; MRI, magnetic resonance imaging; DFS, disease-free survival; OS, overall survival; H&E, hematoxylin and eosin.

Between August 2019 to September 2021, we screened 22 patients. 17 patients had ccRCC shown by biopsy, and these patients were enrolled on the study to receive neoadjuvant cabozantinib for 12 weeks (Extended Data Fig. 1a). The median age of the patients was 56 years (range: 41–84 years) and 82.4% male (Extended Data Table 1). The Eastern Cooperative Oncology Group (ECOG) performance status for all patients was 0. After completion of 12 weeks of treatment and 4 weeks wash-out, 16 patients underwent nephrectomy (Fig. 1a). One patient refused surgery due to personal reasons and received additional systemic treatment. Six patients (35%) experienced a partial response (PR), and 11 patients (65%) had stable disease (SD) (Fig. 1b). All patients had tumor reduction after treatment (100% clinical benefit rate), and there was no progression of disease while on cabozantinib. The median reduction of primary renal tumor size was 26% (range: 8–42%) (Fig. 1c-e and Extended Data Table 1). The one patient who was deemed to be unresectable at the time of enrollment because of the need for multiple adjunctive organ removal became resectable by the end of treatment (Fig. 1f). Two patients were converted from radical to partial nephrectomy (Fig. 1f). The downstaging of patient tumors was decided by a tumor board. The most common adverse events (AEs) from systemic therapy were diarrhea, nausea, fatigue, hypertension, anorexia, and palmar-plantar erythrodysesthesia syndrome which are summarized in Extended Data Table 2. No treatment grade 4 or 5 AEs related to cabozantinib, or surgery occurred. Intraoperatively, we did not experience any increased difficulty in completing surgery. In fact, there seemed to be an increased desmoplastic reaction around the tumor, which facilitated partial nephrectomy. Postoperatively, no surgical complications related to the drug were noted. Grossly (macroscopically), tumors showed variable degrees of tumor necrosis and hyalinization (Fig. 1g). Histologically (microscopically), tumors also demonstrated variable degrees of chronic inflammation, parenchymal and perivascular hyalinization, and necrosis (Fig. 1h). The pathologic response rate is directly proportional to the extent of therapy related changes. The patients that had the most extensive therapy related changes (70–90%) had better pathologic response compared to patients that minimal therapy related changes (10–15%) (Extended Data Table 3).

To assess how this treatment altered long term outcomes of patients, we first assessed disease-free survival (DFS). The median follow-up for 17 treated patients is 25 months. The one-year DFS was 82.4% (95% CI = 54.7% – 93.9%) (Fig. 1i, left). The one-year overall survival (OS) was 94.1% (95% CI = 65% – 99.1%) (Fig. 1i, right). Three patients were deceased at the time of analysis (1 due to progression of RCC, 1 to COVID and 1 from an unknown cause). Overall, these data indicate that cabozantinib was clinically active and safe in the neoadjuvant setting in patients with locally advanced non-metastatic ccRCC, and in some cases allowed surgery for patients with unresectable disease, or de-intensification of the surgical approach.

### Plasma cell-free DNA (cfDNA), cytokines, and radiomic features prior to and during treatment correlate with tumor response

**Extended Data Fig. 2|:**
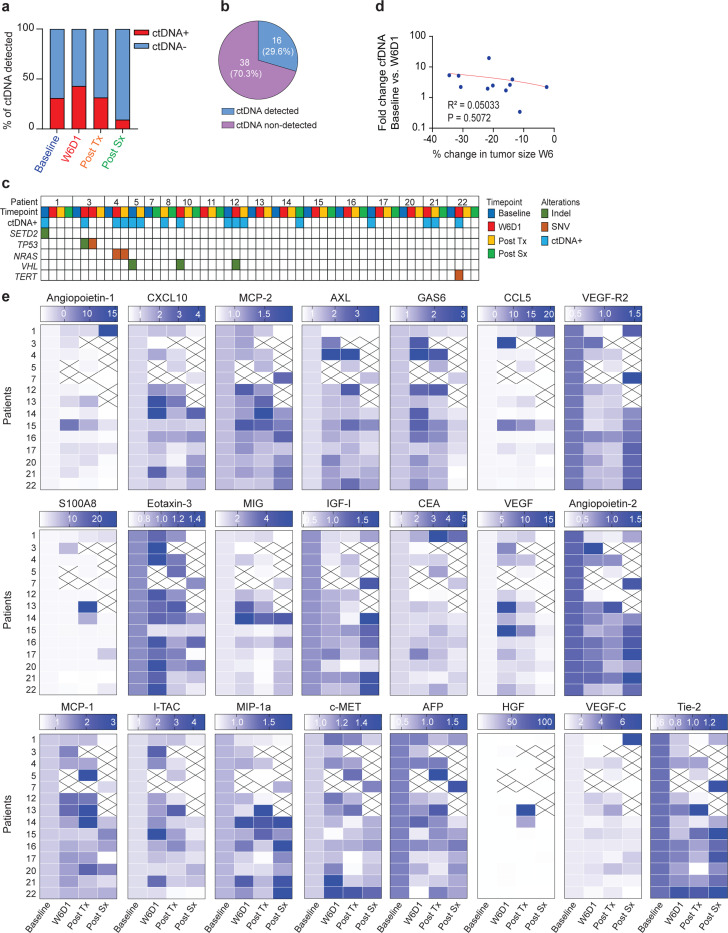
ctDNA and cytokines expressed in plasma. **a**, ctDNA detection for each timepoint: Baseline, 30.8% (4/13); W6D1, 42.9% (6/14); Post Tx, 31.3% (5/16); and Post Sx, 9.1% (1/11). **b**, Percent of ctDNA detected in 16 samples but not in 38 samples. **c**, Oncomap shows alterations identified in ctDNA at four timepoints. **d**, Correlation of cfDNA at W6D1 timepoint and the percent change in tumor size at week 6. **e**, Heatmap of 22 cytokines expression at each timepoints. X indicates samples were not available. cfDNA, cell-free DNA; ctDNA, circulating tumor DNA; W6D1, week 6 day 1.

**Fig. 2|:**
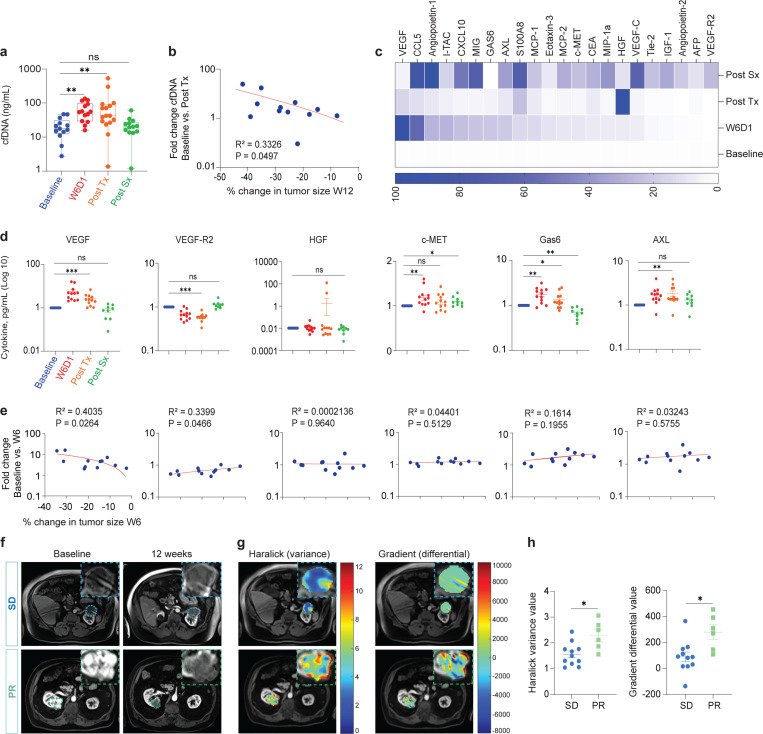
Plasma biomarkers in patients with ccRCC after cabozantinib treatment. **a**, cfDNA (ng/mL) was measured at baseline, W6D1, Post Tx, Post Sx. Statistical analysis resultant from Wilcoxon matched-pair signed rank test is shown. Baseline vs. W6D1 (**, P=0.0098) or Post Tx (**, P=0.0049). **b**, Correlation of cfDNA at Post Tx timepoint and the percent change in tumor size at week 12. **c**, Heatmap of 22 cytokines expression at four timepoints. **d**, Measurement of VEGF, VEGF-R2, HGF, c-MET, Gas6, and AXL at 4 timepoints. Wilcoxon matched-pair signed rank test was used for the analysis. VEGF, baseline vs. W6D1 or Post Tx (***, P=0.0005), Post Sx (ns, P=0.2500). VEGF-R2, baseline vs. W6D1 (***, P=0.0010), Post Tx (***, P=0.0005), or Post Sx (ns, P=0.0742). HGF, baseline vs. W6D1 (ns, P=0.9658), Post Tx (ns, P=0.7910), or Post Sx (ns, P=0.3594). c-MET, baseline vs. W6D1 (**, P=0.0049) or Post Tx (ns, P=0.1763), or Post Sx (*, P=0.0195). Gas6, baseline vs. W6D1 (**, P=0.0024), Post Tx (*, P=0.0342), or Post Sx (**, P=0.0039). AXL, baseline vs. W6D1 (**, P=0.0034), Post Tx (**, P=0.0093), or Post Sx (ns, P=0.0742). **e**, Correlation of VEGF, VEGF-R2, HGF, c-MET, Gas6, and AXL at W6D1 and percent change in tumor size at week 6. **f)** Representative arterial T1W MRI images of SD and PR patients at baseline and 12 weeks. Blue and cyan dashed lines represent tumor. **g)** MRI images of SD and PR patients showing radiomic feature map overlays at baseline using Haralick and Gradient measurements. **h)** Summary data of Haralick and Gradient features in SD and PR patients. The Mann-Whitney test was used for the analysis. Data are presented as mean ± SEM. SD vs. PR (*, P=0.0103) and (*, P=0.0145) for Haralick and Gradient measurements, respectively. W6D1, week 6 day 1; cfDNA, cell-free DNA; VEGF, vascular endothelial growth factor; c-MET, mesenchymal-epithelial transition factor; VEGF-R2, vascular endothelial growth factor-receptor 2; HGF, hepatocyte growth factor.

Having established neoadjuvant cabozantinib as an effective means to reduce tumor burden in some patients, we wanted to know if serum markers could be used to identify which patients might respond better or worse. Plasma cfDNA and circulating tumor DNA (ctDNA) have been described as a liquid biopsy in RCC to identify tumor variants and its role in cancer detection, prognosis, and clinical outcomes^16,17^. Here, plasma cfDNA was isolated from 17 patients with ccRCC collected at four different timepoints: baseline, week 6 day 1 (W6D1), post-treatment (Post Tx) and post-surgery (Post Sx). As shown in Fig. 2a, cfDNA concentrations were significantly increased in W6D1 and Post Tx timepoints compared to the baseline. In addition, ctDNA with mutations in *SETD2, TP53, NRAS, VHL*, and *TERT* was detected in 29.6% of plasma samples (Extended Data Fig. 2a-c and Extended Data Table 4). Spearman correlation analysis showed that cfDNA concentrations Post Tx were significantly correlated with the change in tumor size at week 12 but not week 6 (Fig. 2b and Extended Data Fig. 2d). These data indicate that cfDNA is a strong marker of response to cabozantinib, and as early as 6 weeks significant increase is detected, suggesting future large-scale studies should incorporate use of the marker to determine if it might allow early decisions about continuing or aborting neoadjuvant therapy.

Next, we looked in plasma for changes in expression of 22 cytokines and protein markers previously found related to cabozantinib response^18^. We measured these markers at baseline, W6D1, Post Tx, and Post Sx in duplicate using multiplex enzyme-linked immunosorbent assay (ELISA). Summary heatmaps show differential expressions of the 22 biomarkers (Fig. 2c and Extended Data Fig. 2e). We found that plasma concentrations of VEGF, c-MET, Gas6, and AXL significantly increased during cabozantinib treatment, but not HGF (Fig. 2d and Supplementary Fig. 1). On the other hand, the plasma concentrations of VEGFR2 significantly decreased following treatment with cabozantinib (Fig. 2d). The Spearman correlation coefficients were calculated to determine the association between biomarker levels and percent change in tumor size according to RECIST. We found a significant correlation between plasma VEGF and VEGFR2 biomarkers and the percent change in tumor size at week 6 (Fig. 2e). These data indicate that cfDNA and cytokines correlated with specific changes in tumor growth as early as 6 weeks after beginning treatment and imply that future clinical investigation of this neoadjuvant therapy should include these plasma markers to possible allow early notification of treatment efficacy.

Magnetic resonance imaging (MRI) was used to monitor tumor response to neoadjuvant cabozantinib. In Fig. 2f, we showed arterial phase T1-weighted (T1W) MRI images of SD and PR patients at baseline and 12 weeks. For the SD patient, the tumor was stable in size. In comparison, PR patients at 12-week MRI scan showed dramatic decreased of tumor size compared to the baseline. Computationally derived radiomic features from MRI might quantify differential imaging signatures at baseline associated with response to treatment^19–21^. A set of radiomic features were derived from T1W MRI at baseline and evaluated for differences in SD and PR patients (Fig. 2g). We found that radiomic measurements (specifically Haralick and Gradient features) were significantly higher in PR patients compared to SD patients (Fig. 2h). These features quantify underlying tumor heterogeneity, and our results suggest that tumors that would partially respond to neoadjuvant cabozantinib tend to have a relatively higher heterogenous pattern at baseline compared to those that would result in SD at 12 weeks. Overall, these data indicated that several blood and radiometric features collected before and at early timepoints during neoadjuvant therapy may be predictive of response to therapy and should be analyzed in future trials to determine their predictive strength.

### CD8+ T cells become activated in the peripheral blood during cabozantinib treatment

**Extended Data Fig. 3|:**
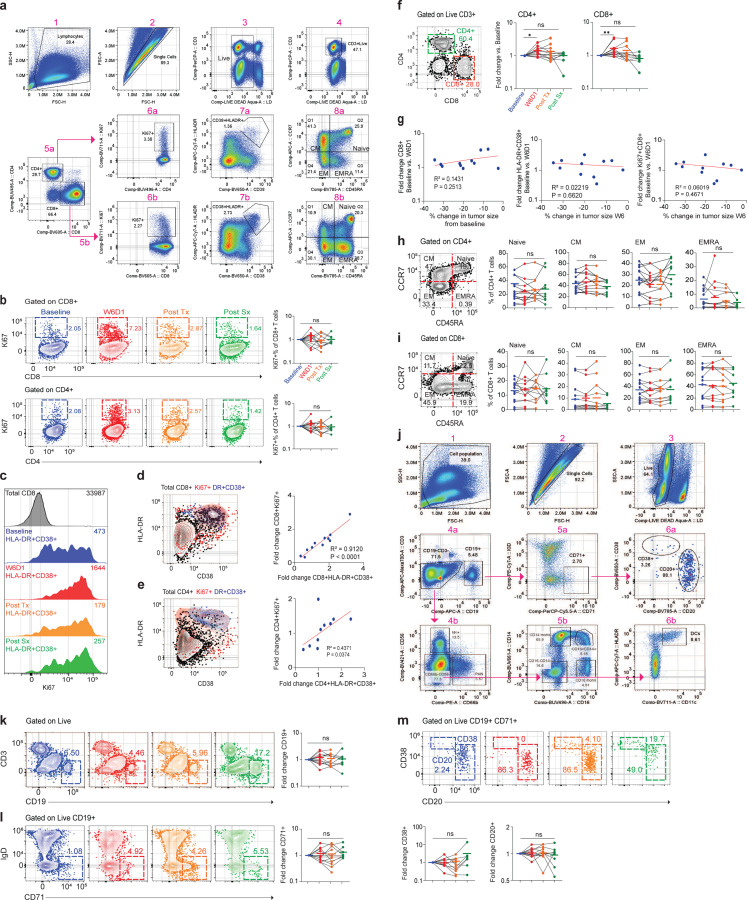
Flow cytometry characterization of immune cells in patient’s peripheral blood after cabozantinib treatment. **a**, Gating strategy to identify Ki67+, HLA-DR+ CD38+ and memory subsets (CD45RA and CCR7) on CD4+ and CD8+ T cells. **b**, Ki67+ expression of CD8+ or CD4+ T cells. Peripheral blood was analyzed by flow cytometry. Flow plots are gated on CD8+ or CD4+ T cells and the expression of Ki67+ cells are displayed in colors. **c**, Histogram plots show HLA-DR+ and CD38+ T cells expression of Ki67 at four timepoints. Total CD8+ T cells are shown as a control. **d** and **e**, Flow plots show HLA-DR+ and CD38+ T cells express Ki67+ in both CD4+ and CD8+ T cells. Overlayed of total CD4+ or CD8+ T cells (blue) that were positive for Ki67 (red) relative positive in the HLA-DR+ and CD38+ cells (purple). Correlation of HLA-DR+ and CD38+ T cells vs. Ki67+ in both CD4+ and CD8+ T cells. **f**, FACS plot shows cells gated on live and CD3+. The percentage of live PBMCs of CD4+ and CD8+ T cells. **g**, Correlation of CD8+, HLA-DR+ CD38+ and Ki67 of CD8+ T cells at W6D1 timepoint and the percent change in tumor size at week 6. **h**, Canonical CD4 memory subsets over treatment period. Cells were gated on CD4 and analyzed for expression of CD45RA and CCR7. **i**, Canonical CD8 memory subsets were gated on CD8, then CD45RA and CCR7 expression. **j**, Gating strategy to identify B-cells, monocytes, and dendritic cells. **k** - **m**, Expression of CD19 (k), CD71 (l), CD38 and CD20 (m), in the peripheral blood analyzed by flow cytometry. For each summary plot, baseline was set as the untreated level for each patient and fold change in these cells expressed versus this timepoint. Summary of P values are in the Supplementary Tables 6 and 7.

**Fig. 3|:**
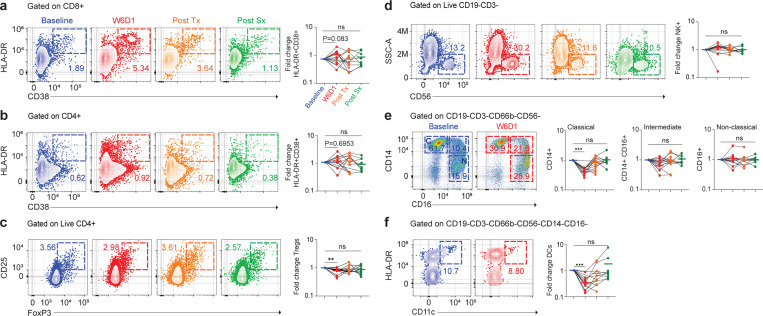
Comprehensive analysis of immune cells in patient’s peripheral blood. **a**, Ki67+ of CD8+ T cells expression in the peripheral blood analyzed by flow cytometry. Flow plots are gated on CD8+ T cells and the expression of HLA-DR+CD38+ cells are displayed in colors. Summary plots show fold change in HLA-DR+CD38+ of CD8+ T cells after cabozantinib treatment. **b**, HLA-DR+CD38+ of CD4+ T cells expression gated on CD4+ T cells. **c**, Tregs expression in the peripheral blood. Flow plots are gated on CD4+ T cells. **d**, Flow plots showing expression of NK+ cells. **e**, Monocyte subsets based on surface markers CD14 and CD16 were identified by flow cytometry in a representative patient. Flow plots are gated on CD19-CD3-CD66b-CD56- cells. Classical monocytes are CD14++CD16-; intermediate monocytes are CD14++CD16+; and non-classical monocytes are CD14+CD16+. **f**, Expression of DCs (MHC-II+CD11c+) in the peripheral blood. Baseline was set as the untreated level for each patient and fold change in these cells expressed versus this timepoint. Statistical analysis resultant from Wilcoxon pair sign rank test was used for the analysis. Summary of P values are in the Supplementary Table 7.

Because several preclinical studies have found cabozantinib alters anti-tumor immunity^12–15^, and neoadjuvant therapies may provide long term-survival benefits by providing long-lasting immunity^8,22,23^, we wanted to see if there were any signs of immune activation in blood of patients. We collected blood from patients at baseline, W6D1, Post Tx, and Post Sx and performed a comprehensive immune cell analysis for each patient (Extended Data Fig. 1a and 3a). We first measured two markers indicating T cell activity, expression of Ki67 to indicate recent proliferation, and co-expression of the markers HLA-DR and CD38 which indicates recent activation and have been associated with response to immunotherapy^24–27^. After the first cycle (W6D1) of cabozantinib, there was a trend (P=0.0830) towards expansion of CD8+ T cells expressing HLA-DR+CD38+ in the blood with at least 1.6-fold increase compared to the baseline timepoint (Fig. 3a). Similarly, there was a trend towards an increase in the proportion of CD8+ T cells expressing Ki67+ at W6D1 (Extended Data Fig. 3b), and a strong overlap between Ki67+ and HLA-DR+CD38+ cells (Extended Data Fig. 3c-e). However, by the Post Tx and Post Sx timepoints, these differences were decreased (Fig. 3a and Extended Data Fig. 3b). In comparison, CD4+ T cells had no increase in HLA-DR+CD38+ (Fig. 3b) or expression of Ki67 (Extended Data Fig. 3b). However, in the CD4+ compartment, we found that Tregs were depleted at W6D1 and Post Tx timepoints compared to the baseline (Fig. 3c). In addition to increased activation, the proportion of CD4+ and CD8+ T cells in the blood were significantly increased at W6D1 and decreased at Post Sx timepoint (Extended Data Fig. 3f). Next, we assessed whether each patient’s percent change in tumor size was correlated with T cell activation by RECIST criteria. We identified that total CD8+, HLA-DR+CD38+ and Ki67+ of CD8+ T cells expression was not correlated with the percent change in tumor size (Extended Data Fig. 3g). Similarly, the breakdown of naïve, T_cm_, T_em_, and T_emra_ of CD4+ and CD8+ T cell subsets (CD45RA and CCR7 expression) were not significantly different at any timepoint during cabozantinib treatment (Extended Data Fig. 3h, i).

We next turned our attention to other immune populations in the blood and looked at changes in natural killer (NK) cells, B cells, monocyte, and dendritic cells (Extended Data Fig. 3j). To determine whether activated B cells were expressed after cabozantinib treatment, we looked at several B cell sub-populations (CD19, CD20, CD38, and CD71) but generally no significant differences were observed (Extended Data Fig. 3j-m). There was a slight increase in NK cells during W6D1 of treatment but no other timepoints compared to the baseline (Fig. 3d). In comparison to these minor changes, there were much more significant decreases in myeloid populations. Human monocytes are divided into three subsets: classical (CD14+CD16-), intermediate (CD14+CD16+), and non-classical (CD14-CD16+). We found that classical monocytes were significantly reduced in every patient during W6D1 of cabozantinib treatment and slight increased for some patients at Post-Tx and -Sx timepoints (Fig. 3e). Intermediate monocytes were also decreased during W6D1 of treatment, while there was not significantly different of non-classical monocytes at any timepoints (Fig. 3e). Moreover, we also assessed the antigen presenting cells (APCs) in the blood. We found that dendritic cells (DCs) (HLA-DR+CD11c+) expression levels in all patients significantly decreased in W6D1 (Fig. 3f). We also see similar results with the absolute values for each FACS marker; summary plots are in the Supplementary Fig. 2a-f and Supplementary Table 6. Together, these data indicate that cabozantinib has several effects on the immune system of patients. While it clearly has a strong depleting effect on the myeloid compartment, the effect on CD8+ T cells is more in line with immunotherapy, where a consistent increase in T cell proliferation is observed on the initial treatment^28^.

### CD8+ T cell infiltration into tumors is increased in patients receiving cabozantinib

**Fig. 4|:**
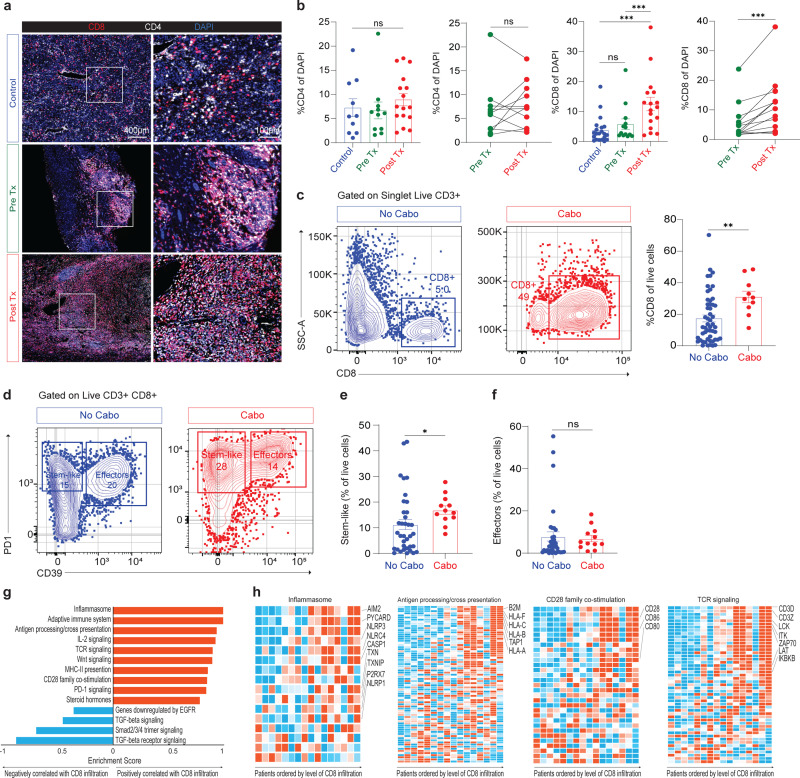
T cells activation in patient’s tumors. **a**, Immunofluorescence tumor images of CD4 (white), CD8 (red), and DAPI (blue) in control (historical data), Pre Tx (biopsy), and Post Tx (cabozantinib treatments) groups. **b**, Summary quantitative immunofluorescence data of CD4 and CD8 percent of DAPI in the control (CD4, n=10 and CD8, n=21), Pre Tx (n=12), and Post Tx (n=16) groups. Statistical analysis resultant from Mann-Whitney test is shown. Data are presented as mean ± SEM. Control vs. Post Tx (***, P=0.0002). Wilcoxon matched pair signed rank test was used for Pre Tx vs. Post Tx (***, P=0.0005). **c**, Representative plots showing activated CD8+ T cells in human ccRCC T3N0M0 tumors. Blue color represents historical published data (No Cabo) and red color represents tumors of patients treated with cabozantinib (Cabo). Summary of CD8+ T cells in historical data (No Cabo, n=52) and Cabo tumors (Cabo, n=10). Statistical analysis resultant from Mann-Whitney test is shown. No Cabo vs. Cabo (**, P=0.0036). **d**, Flow cytometry plots showing expression of stem-like and effectors in historical data and cabozantinib tumors. **e** and **f**, Summary of stem-like (**e**) and effector cells (**f**) in historical data (n=36) and Cabo tumors (n=12). Statistical analysis resultant from Mann-Whitney test is shown. No Cabo vs. Cabo (*, P=0.0161) in **e. g**, Results of GSEA showing pathways that are negatively and positively correlated with CD8+ T cell infiltration. **h**, Summary of heat maps showing enriched gene sets in inflammasome, antigen processing/cross presentation, CD28 family co-stimulation, and TCR signaling of patients ordered by level of CD8+ T cell infiltration. ns, not significant; SEM, standard error of the mean; ccRCC, clear cell renal cell carcinoma; DAPI, 4’,6-diamidino-2-phenylindole; GSEA, gene set enrichment analysis.

We were next interested in how cabozantinib changed the tumor immune microenvironment. To do this we measured total CD4+ and CD8+ T cells in tumor using multiplex immunofluorescence (mIF). We compared three groups: 1) Control tumors untreated patients and matched for stage of disease from our historical published data^29,30^, 2) Pre Tx biopsy from patients before cabozantinib treatment and 3) Post Tx tumor tissues from this trial after nephrectomy. We found there was no significant difference in the percentage of CD4+ T cells of total DAPI+ cells between any of these groups (Fig. 4a, b). In comparison, CD8+ T cells infiltration significantly increased (8.782 ± 2.282) in patients treated with cabozantinib compared to the control and Pre Tx groups (Fig. 4a, b).

**Extended Data Fig. 4|:**
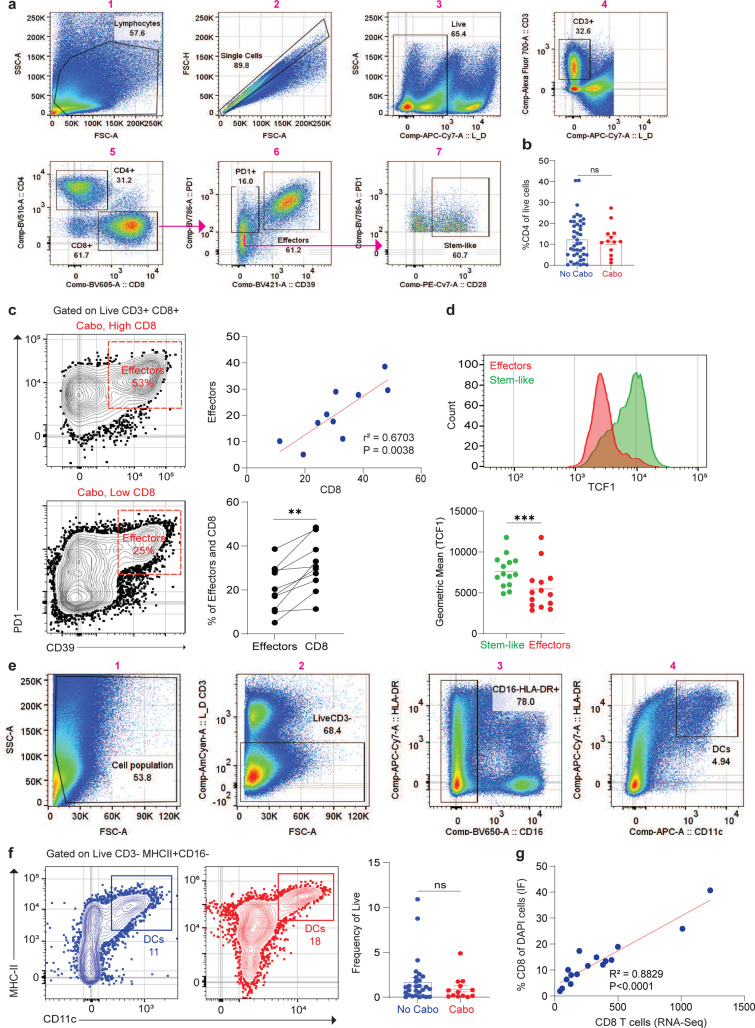
**a**, Gating strategy from a representative patient to identify effectors (PD1+CD39+) and stem-like (PD1+ CD39- or PD1+CD28+) of CD8 T cells. **b**, Summary of CD4+ T cells in historical data (n=52) and Cabo tumors (n=10). Mann-Whitney test was used for the analysis. Data are presented as mean ± SEM. ns, not significant. **c**, Flow cytometry plots of high and low CD8-infiltrated kidney tumors in cabozantinib treatments. Summary data showing the correlation between CD8 T cells and effectors cells. Wilcoxon matched pair signed rank test was used for the analysis. Effectors vs. CD8 (**, P=0.0020). **d**, FACS and summary plots of stem-like and effector cells in TCF1 expression. Wilcoxon pair test was used for stem-like vs effectors (***, P=0.0002). **e**, Gating strategy to identify the expression of DCs (MHC- II+CD11c+). **f**, Flow cytometry analysis of DCs in historical and cabozantinib tumors. Summary of DCs expression in historical data (n=28) and Cabo tumors (n=13). Statistical analysis resultant as described in **b. g**, Summary data showing the Spearman correlation of CD8+ T cells from RNA-Seq and CD8+ percent of DAPI from immunofluorescence.

To further confirm this observation, we performed flow cytometry on resected tumor from the trial and compared to historical published flow cytometry data from patients with T3N0M0 who had previously undergone nephrectomy^29,30^ (Extended Data Fig. 4a). Blue color (No Cabo) represents historical published data in disease matched patients, and red color (Cabo) represents tumors of patients from this trial treated with cabozantinib. Similar observation by IF, the percentage of CD4+ T cells of live cells in the historical data were unchanged compared to the cabozantinib treatment group (Extended Data Fig. 4b). In contrast, CD8+ T cells infiltration significantly increased 3-fold in patients treated with cabozantinib compared to the historical data (Fig. 4c).

Previously, we have shown that highly infiltrated tumors had a distinct population of both stem-like cells and terminally differentiated cells^28,29^. These cells are essential to mediating response to PD1 blockade and maintaining the T cell response against cancer^31–35^. We found that cabozantinib patients who have high CD8+ T cell infiltration also have high stem-like (PD1+CD39-) expression compared to the historical data (Fig. 4d, e). However, there were no differences in the CD39 terminally differentiated or effector cells when comparing patients with or without cabozantinib treatments (Fig. 4f). We also identified that patients with high CD8+ T cells infiltration are strongly correlated with CD39 terminally differentiated cells (Extended Data Fig. 4c). TFC1 expression is significantly higher in stem-like cells compared to the effector’s cells (Extended Data Fig. 4d). In addition, we measured the tumor-infiltrating APCs in the tumor by MHC-II+ and CD11c+ (Extended Data Fig. 4e). The levels of DCs were unchanged between historical samples and those receiving cabozantinib treatment (Extended Data Fig. 4f).

To learn more about the transcriptional events in the tumor microenvironment that might correlate with this increased CD8+ T cell infiltration, we performed RNA-Seq on formalin-fixed paraffin-embedded (FFPE) tissue at the time of surgery and correlated transcriptional pathway changes with CD8+ T cell infiltration (Extended Data Fig. 4g). The pathways most correlated with CD8+ T cell infiltration included the inflammasome, antigen processing and cross presentation and several pathways related to T cell receptor signaling and co-stimulation (Fig. 4g). In prior studies, we have reported that CD8+ T cells require activated APCs expressing co-stimulatory molecules in the tumor microenvironment for generation of effector cells^30^. We found high enrichment of many genes from the inflammasome pathway correlated with CD8+ T cell infiltration that are involved in sensing danger signals, activating APCs and many pathways related to antigen cross presentation (Fig. 4h). The data provides new information showing that cabozantinib treatment induces CD8+ T cell infiltration to over 10% of the total tumor. A level far above the 2.2% level we previously found to correlate with improved survival^29^.

### Cabozantinib induces broad generation of immune niches containing TCF1+ stem-like CD8+ T cells

**Extended Data Fig. 5|:**
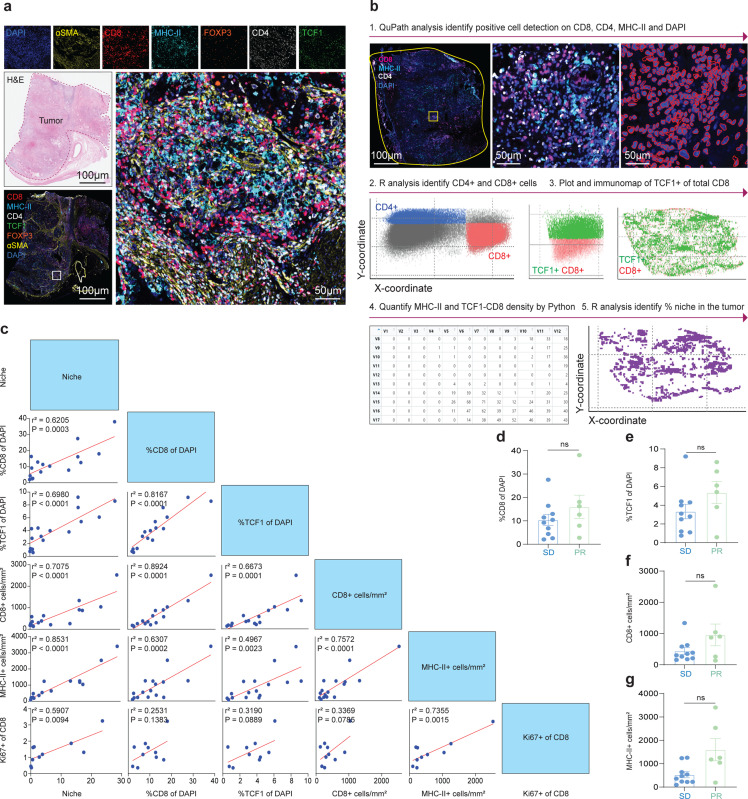
Quantitative imaging analysis of patient with and without cabozantinib treatment. **a**, H&E tumor image of a cabozantinib treated patient. The tumor was highlighted in red dashed line. Whole slide and single channel immunofluorescences of CD8 (red), MHC-II (cyan), CD4 (white), FOXP3 (orange), aSMA (yellow), and DAPI (blue). **b**, Workflow for immunofluorescence imaging analysis and immunomap creation. Single channel immunofluorescence images are imported into QuPath software. CD8, CD4, MHC-II, and DAPI objects are identified in the respective channel images. The XY location of each object is exported. R analysis was used to identify CD4+ or CD8+ cells. The TCF-1+ intensity is measured inside the CD8+ objects. These parameters were used to calculate the MHC-II+ cell density. The distance of each CD8+ object was measured to its nearest MHC-II+ neighbor and to create immunomaps using custom R and Python scripts. **c**, Correlation matrix summary data for quantitative immunofluorescence of cabozantinib tumors. The percentage of CD8+ and TCF1+ of DAPI, CD8+ T cell infiltration, MHC-II+ cell infiltration, immune niche in the tumors, and Ki67+ of CD8 in PBMCs were compared to each subgroup. For example, the percent of CD8+ of DAPI was correlated with percent of immune niche. **d-g**, Summary data comparing SD and PR patients with percent of CD8+ of DAPI (**d**), percent of TCF1+ of DAPI (e), CD8+ T cell infiltration (**f**), MHC-II+ cell infiltration (**g**) in cabozantinib treated patients. Statistical analysis resultant as described in Fig. 5a. ns, not significant (**d-g**). PR, partial response; SD, stable disease; H&E, hematoxylin and eosin; DAPI, 4’,6-diamidino-2-phenylindole

**Fig. 5|:**
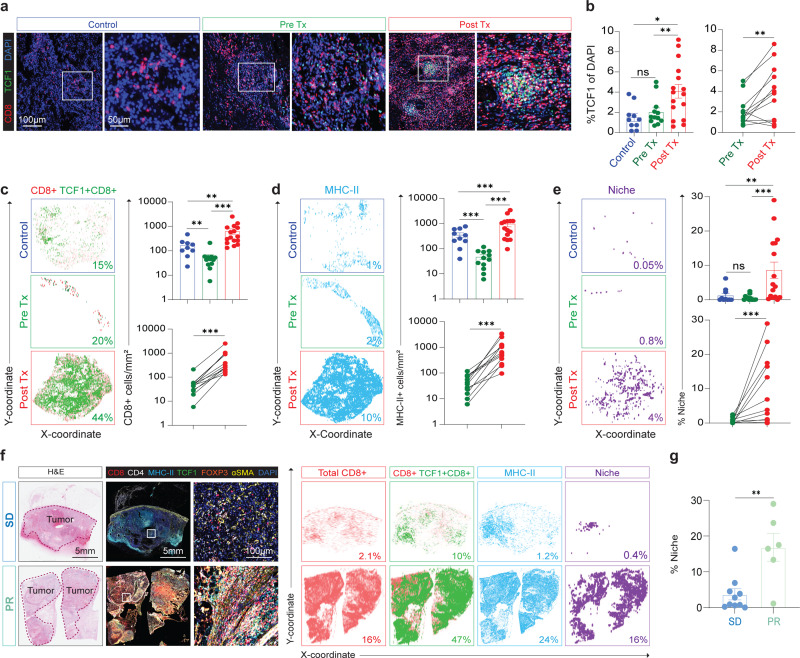
Cabozantinib treatments active CD8+ T cells in patient’s tumors. **a**, Immunofluorescence tumor images of representative patients in control, Pre Tx, and Post Tx groups. CD8 (red), TCF1 (green), and DAPI (blue). **b**, Summary data comparing TCF1 percent of DAPI in Control (Blue, n=10), Pre Tx (Green, n=12), and Post Tx (Red, n=16) groups. The Mann-Whitney test was used. Data are presented as mean ± SEM. Control vs. Post Tx (*, P=0.0122). Wilcoxon matched pair signed rank test was used for Pre Tx vs. Post Tx (**, P=0.0093). **c** and **d**, Quantitative analysis of immunofluorescence of CD8+ T cells, TCF1+ of CD8+T cells and MHC-II+ cells. Spatial plots show each of these subsets are found in the tumor and summary plots show the proportion of these cells in tumors of representative patients who received cabozantinib compared to the Control and Pre Tx groups. Statistical analysis resultant as described in **b**. For **c**, Control vs. Pre Tx (**, P=0.0056) and Control vs. Post Tx (**, P=0.0018). Wilcoxon matched pair test was used for Pre Tx vs. Post Tx (***, P=0.0005). For **d**, Control vs. Pre Tx (***, P=0.0001) and Control vs. Post Tx (***, P=0.0001). Wilcoxon matched pair test was used for Pre Tx vs. Post Tx (***, P=0.0005). **e**, Niches were defined as regions containing ≥ 16 MHC-II+ cells and ≥ 4 TCF1+CD8 T cells in the same area of the whole tumor tissue. Spatial and summary plots of niches in representative patients with cabozantinib treatments versus the Control and Pre Tx groups. Control vs. Pre Tx (ns, not significant); Control vs. Post Tx (**, P=0.0041). The Wilcoxon pair test was used for Pre Tx vs. Post Tx (***, P=0.0010). **f**, SD and PR patients with high and low CD8+ T cell infiltration. H&E images of the whole slide. The tumor is outlined in red. Whole slide mIF images consist of CD8+ (red), MHC- II (cyan), CD4 (white), FOXP3 (orange), αSMA (yellow), and DAPI (blue). Immunomaps illustrating regions of CD8+ (red), TCF1+ of CD8+ (green), MHC-II+ (cyan), and immune niche cell density in tumors. **g**, Summary data comparing SD and PR patients with percent of niche. Statistical analysis resultant as described in **a**. SD vs. PR (**, P=0.0075). SEM, standard error of the mean; PR, partial response; SD, stable disease; TCF1, T cell factor 1; H&E, hematoxylin and eosin; DAPI, 4’,6-diamidino-2-phenylindole.

In prior work, we had described the presence of an immune niche in tumors^28,29^. These niches are made up of APCs that co-localize with stem-like TCF1+ CD8+ T cells and correlate with survival after surgery and response to immunotherapy in patients with RCC^28,29^. Given the large increase we saw in CD8+ T cell infiltration, we next examined how cabozantinib altered immune niche formation in kidney tumors. To do this we analyzed surgically resected tumors from 16 patients who received neoadjuvant cabozantinib by mIF (Extended Data Fig. 5a) and compared it to our historical IF data and Pre Tx biopsy samples. Patients who received cabozantinib had significantly more TCF1+ in their tumors compared to both the historical and Pre Tx biopsy data (Fig. 5a, b). We generated quantitative maps of each tumor section to learn how immune niche formation was altered by treatment with cabozantinib. The whole tumor slide was quantified for CD4, CD8, MHC-II, and DAPI markers and detected the XY location for each cell. The immunomaps in Fig. 5c-e show the XY location of all CD8+ T cells (red), TCF1+ CD8+ T cells (green), and MHC-II+ cells (blue). There were significantly more TCF1+ CD8+ T cells and MHC-II+ cells in the cabozantinib tumors compared to untreated tumors and Pre Tx biopsy (Fig. 5c-e). In line with our previous studies^28,29^, we found the number of CD8+ T cells correlate strongly with the amount of MHC-II+ and TCF1+ CD8+ T cells in the tissue (Extended Data Fig. 5c). We also identified immune niche (purple) regions containing ≥ 16 MHC-II+ cells and ≥ 4 TCF1+ CD8+ T cells per mm^2^ within the same area of the whole tumor tissue (Extended Data Fig. 5b). Importantly, we found that cabozantinib substantially increased the proportion of the tumor covered by these niches. The proportion of the tumor that was made up with immune niche strongly correlated with the proportion of MHC-II+ and TCF1+ cells in the tumor.

In this study, 6 patients experienced PR and 11 patients had SD. In all these patients there was a very high percentage of CD8+ T cells compared to control patients (Fig. 5f). Patients who underwent PR had a trend towards having more CD8+ T cells, TCF1+ stem-like, CD8+ and MHC-II+ cells density compared to SD patients but was not significant (Extended Data Fig. 5d-g). Most importantly, however, patients who had PR had significantly higher levels of immune niches in their tumor compared to patients with stable disease (Fig. 5f, g). In summary, these results indicate that cabozantinib treatment in patients with ccRCC induces high CD8+ T cells in tumors, and importantly increases the presence of TCF1+ stem-like CD8+ T cells.

## Discussion

In this prospective trial, we present the first findings to demonstrate neoadjuvant cabozantinib as a safe, effective oral regimen, and highly effective at reducing tumor size in a 12-week treatment prior to nephrectomy in non-metastatic, clinical stage T3 and T4 ccRCC. In total, 6 of 17 (35%) patients experienced a partial response. All patients had clinical benefit with no signs of progression prior to partial or radical nephrectomy. There were no major toxicities or surgical complications from pre-treatment cabozantinib, one patient became eligible for surgery who previously had unresectable disease, and 2 patients were able to have nephron sparing surgery in place of radical nephrectomy.

Since 2009, several neoadjuvant clinical trials have been conducted in patients with ccRCC using VEGF and other TKIs. These trials have generally found similar results to what we report here; reduced tumor burden allowing unresectable cases to undergo nephrectomy, or de-intensification of the surgical approach allowing partial instead of radical nephrectomy^36–40^. Most relevant to this trial are two phase 2 trials from 2015 and 2019, studying neoadjuvant axitinib in locally advanced, non-metastatic ccRCC (cT2a-T3bN0M0). Similar to the trial reported here, these drugs were well tolerated with no grade 4–5 AEs and demonstrated a primary median size reduction of 18–28%, comparable to our findings^41–43^. In comparison to these TKI trials, two separate phase 2 trials, neoadjuvant nivolumab administered to patients with clinically localized high-risk RCC did not demonstrate significant size reduction^44,45^ but does have modest survival benefit in the adjuvant setting^46^. In both these trials, there was evidence of a pathologic response in select patients characterized by increased immune cell infiltration. These observations set ccRCC apart from what has been observed in other immune responsive tumors like melanoma and lung cancer^8^, where neoadjuvant immunotherapy seems to have a clear benefit to OS.

The most striking correlative data from this trial was the systemic activation of CD8+ T cells. Every patient had a large increase in CD8+ T cells in their tumor that included both TCF1+ stem-like populations and TCF1-effectors. Importantly, immune niches made up of the stem-like CD8+ T cells and APCs, which we have found in many tumor types and correlate with survival and response to immunotherapy, were increased from pre-treatment levels in every patient. The PR patients who had at least a 30% decrease in tumor size had significantly more of these niches generated. While cabozantinib is not designed as an immunotherapy, its immunomodulatory activities have been demonstrated in preclinical and clinical studies. For example, in a preclinical mouse model of hepatocellular carcinoma (HCC) showed that cabozantinib administration promotes the recruitment of neutrophils and reduced intratumor CD8+ PD1+ T cells and Tregs while enhanced memory/effector T cell proportions in the blood^12^. In clinical trials, patients with metastatic RCC, platinum-refractory urothelial carcinoma (phase 2 trial), triple-negative breast cancer (phase 2 trial), and castration-resistant prostate cancer (phase 1b), all have shown that cabozantinib increased cytotoxic T cells and reduced peripheral MDSCs and regulatory T cells^18,47–49^ similar to what we found in our study. These findings are important because it is well established that patients with a pre-existing T cell response are far more likely to respond to checkpoint therapy. Many kidney cancer patients have essentially no T cells in their tumors at the time of surgery, so the significance of finding every patient having very high T cell responses in the tumor could be a way to prime the immune response against cancer before giving immunotherapy^50^. Therefore, future trials should investigate the combination of cabozantinib with immunotherapy and include cfDNA as a predictive model to determine whether this parameter correlates with patient response and recurrence. In addition, it will be important to determine if CD8 infiltration correlates with response to cabozantinib and immunotherapy in future large trials. Kidney cancer has had some mixed findings where high levels of infiltration have been associated with worse survival, but features like dendritic cells, proliferating T cells, or RNA signatures associated with T cell responses correlate with better survival and better response to some therapies^51–56^. Future trials will need to include detailed analysis of molecular and immune response in patients to help better understand these mixed findings.

Finally, while this trial was not designed with a comparator arm to directly quantitate a survival benefit, only 3 of the 17 patients progressed in the year after surgery. Given the increased immune response seen, there is strong rationale that cabozantinib given prior to surgery may have long lasting effects on survival by stimulating an anti-tumor response that has lasting effects. Based on these data, we believe further investigation of this approach for kidney cancer is strongly supported.

## Methods

### Study Design

This was an open label, single-arm, phase 2 study of neoadjuvant cabozantinib in patients with locally advanced non-metastatic ccRCC. The study was approved by the Institutional Review Board (IRB)/Ethics Committee at Emory University. The trial enrolled patients at the Winship Cancer Institute at Emory University started in August 2019 and was completed in May 2023 (NCT04022343). The Data and Safety Monitoring Committee (DSMC) of the Winship Cancer Institute provided oversight of this study, to ensure that research being conducted by investigators produces high-quality scientific data in a manner consistent with good clinical practice (GCP) and appropriate regulations that govern clinical research. The DSMC reviewed pertinent aspects of the study to assess subject safety, compliance with the protocol, data collection, and risk-benefit ratio. All study participants were kept confidential per institutional guidelines and policies by assigning a random number to each study participant.

Patients were enrolled if they were diagnosed with ccRCC on pre-treatment biopsy of the primary tumor. Patient renal mass consistent with a clinical stage ≥ T3Nx or TanyN+ or deemed unresectable by surgeon. Patients were required to be 18 years of age or older on the day of consent and have an ECOG performance status ≤ 1. Patients needed to have adequate organ and marrow function. No hormonal therapy, chemotherapy, immunotherapy, or any other systemic therapy for a malignancy in the 5 years prior to current study enrollment. Sexually active patients and their partners needed to agree to use medically accepted methods of contraception during the study and for 4 months after the last dose of study treatment.

Patients with ccRCC were enrolled to receive neoadjuvant cabozantinib for 12 weeks before surgical resection. If patients are eligible and want to be part of the study, the patients will participate for up to 3 years. The study consists of 4 treatment periods: 1) A pre-treatment period in which patients consented to undergo screening assessments to be qualified for the study.Blood samples were collected at this time point as baseline. 2) A treatment period in which patients received cabozantinib orally at a starting dose of 60 mg once daily and undergo study assessments. Dose reduction allowed by protocol, cabozantinib was reduced up to 20 mg per day. This period ended at the time of completion of cabozantinib, or when the patients withdraw consent or experienced unacceptable toxicity. A second blood sample was collected at week 6 day 1 and imaging was performed. 3) A post-treatment period in which patients returned to the study site within 14 days after their last dose of cabozantinib to complete end-of-study assessments. There was a minimum of 28 days washout period from the last dose of cabozantinib prior to surgical resection. Patients were assessed for intraoperative and post-operative complications using the universally recognized Clavien Dindo perioperative classification of adverse events^57^. A third blood sample was collected during this period and imaging was performed. 4) A final blood sample for correlative studies was collected after surgery. There was a long-term follow-up period in which patients were followed after surgery.

Patients were excluded from the study if they had evidence of metastatic disease on pre-treatment imaging, known brain metastases or cranial epidural disease, received of any type of cytotoxic, biologic or other systemic anticancer therapy for kidney cancer, or received any other type of investigational agent within 28 days before the first dose of study treatment, concomitant anticoagulation with oral anticoagulants, prothrombin time (PT) or partial thromboplastin time (PTT) test ≥1.3x the laboratory ULN within 14 days before the first dose of study treatment, uncontrolled significant intercurrent or recent illness such as cardiovascular disorders, gastrointestinal (GI) disorders, endotracheal or endobronchial disease, major surgery within 8 weeks before first dose of study treatment, woman become pregnant or lactating females, inability to swallow tablets, previously identified allergy or hypersensitivity to components of the study treatment formulations, diagnosed another malignancy within 2 years before first dose of study treatment, except for superficial skin cancers, or localized, low grade tumors deemed cured and not treated with systemic therapy.

Objective response rate (ORR) at 12 weeks was the primary endpoint of the study which was evaluated using Response Evaluation Criteria in Solid Tumors (RECIST) 1.1 criteria^58^. All tumor measurements were recorded in centimeters. This was obtained after the last dose of cabozantinib before surgical resection while waiting 28 days wash-out period. In addition to week 12 scan, week 6 scan was obtained to rule out rapid progression by independent radiologist review. Only scans at week 12 (prior to surgery) were used for purposes of the primary objective. Secondary outcomes included safety, tolerability, clinical outcome (DFS, OS), surgical outcome and quality of life.

### Plasma collection and circulating-tumor DNA (ctDNA) analysis

Blood samples from the patients were collected in BD Vacutainer EDTA tubes, subsequently double spun, and the isolated plasma was stored at −80°C. The isolated plasma samples were shipped to Guardant Health, Inc. (Redwood City, CA) for sequencing in a CLIA-certified, CAP-accredited facility. All samples were run on the RUO Guardant Reveal powered by GuardantINFINITY Platform, a plasma-only, next-generation sequencing assay for detecting minimum residual disease (MRD). Physical processing of the samples was performed in accordance with a previously described GuardantOMNI assay^59^. The GuardantINFINITY platform captures a larger genomic component of ~800 gene panel compared to the previous generation platform. Additionally, GuardantINFINITY extends beyond the genomic-only panel capabilities by assaying DNA methylation information through a proprietary non-destructive technique, enabling the capture and enrichment of hypermethylated regions of the samples, and subsequently sequencing this along with the genomic partition Illumina’s NovaSeq platform. A proprietary bioinformatics algorithm was trained on both samples from patients with cancer and cancer-free controls; the outcome of the algorithm is a binary classification of the samples (ctDNA-positive or ctDNA-negative) having been determined based on differentiated methylated regions. Variants identified in the ctDNA-positive samples were filtered to remove those with a high-likelihood of deriving from clonal hematopoiesis of indeterminate potential (CHIP) based on internal and external clinical studies.

### RNA-Seq

Consecutive 10 μm sections were prepared from formalin-fixed paraffin embedded (FFPE) blocks and areas of relevant pathology were circled on one slide which had been stained with H&E. The identified areas were macrodissected from the slides and placed into AutoLys M Tubes (ThermoFisher) for deparaffinization. Sequential DNA and RNA isolation from the recovered tissue was performed on a KingFisher Flex using the Applied Biosystems MagMAX FFPE DNA/RNA Ultra Kit (ThermoFisher). RNA quality was assessed using a TapeStation 4200 (Agilent) and 50 nanograms of total RNA was used as input for library preparation using the SMARTer Stranded Total RNA-Seq Kit v2 (Takara Bio) according to the manufacturer’s instructions. RNA-Seq Libraries were validated by capillary electrophoresis on a TapeStation 4200 (Agilent), pooled at equimolar concentrations, and sequenced with PE100 reads on an Illumina NovaSeq 6000, yielding ~50 million reads per sample on average.

### Biomarker assays

Blood for plasma samples was collected at baseline, week 6 day 1, post-treatment, and postsurgery in BD Vacutainer cell preparation tubes and processed into plasma from 17 patients with ccRCC. All plasma biomarkers were measured by AssayGate, Inc. (Ijamsville, MD). Plasma protein levels of AXL, GAS6, c-MET, and IGF-1 were measured by ELISA assay. Eotaxin-3/CCL26, MCP-2/CCL8, MIG/CXCL9, IP-10/CXCL10, I-TAC/CXCL11, CCL2/JE/MCP-1, MIP-1a, RANTES/CCL5, VEGF-A, CEA, AFP, S100A8, HGF, VEGFR-2, VEGF-C, Angiopoietin 1, Angiopoietin 2, and Tie 2 were measured by Luminex Multiplex assay. The duplicate readings were averaged for each standard control and samples and subtract the average zero standard optical density (ELISA) or fluorescent signals (Luminex). Standard curve was created by reducing the data using computer software capable of generating a four-parameter logistic (4-PL) curve fit (ELISA) or 5-PL (Luminex).

### Sample collection, processing, and flow cytometry

Patients with non-metastatic ccRCC were given informed consent for blood collection. Peripheral blood was collected prior to initiation of study therapy (at baseline), week 6 day 1 (+/− 5 days), the completion of treatment prior to surgery and post-surgical resection. Peripheral blood was obtained in glass mononuclear cell preparation tubes (CPT) and processed to cryopreserve peripheral blood mononuclear cells (PBMCs) and plasma.

Patient tumor samples were collected in Hank’s Balanced Salt Solution (HBSS) after underwent partial or radical nephrectomy. The samples were cut into small pieces, digested with DNase I, collagenase P, and dispase cocktail, and then homogenized using a MACS Dissociator. Digested tumor was washed through a 70 μm filter to get a single cell suspension. Red blood cells were lysed using H_2_O and 1.8% NaCl, fat was removed using 44% Percoll/56% RPMI gradient, and samples were cryopreserved in 90% FBS and 10% DMSO at −80°C^29,30^.

Tumor samples for multiplex immunofluorescence (mIF) and RNA-Seq were formalin fixed and embedded in paraffin blocks by Department of Pathology - Emory University. Unstained and H&E-stained slides of formalin fixed paraffin embedded (FFPE) blocks were obtained from the Cancer Tissue and Pathology Core Facility of Winship Cancer Institute of Emory University.

Single cell suspensions from processed human tumor samples and peripheral blood were stained with antibodies listed in Extended Data Table 5. Live/dead staining was done using fixable near-IR or aqua dead cell staining kit (Invitrogen)^4^ Cells were permed using the FOXP3 Transcription Factor Staining Buffer Set (eBioscience) for 45 minutes at 4°C and stained with intracellular antibodies in permeabilization buffer for 30 minutes at 4°C. Samples were acquired on Cytek Aurora instrument and analyzed using FlowJo (v10) software.

### Multiplex immunofluorescence (mIF)

FFPE tissue sections of 5 μm thickness were used for immunofluorescence staining. Sections were deparaffinized in xylene and rehydrated by serial passage through graded concentrations of ethanol.

mIF staining was performed with the Opal Polaris 7-color fluorescence immunohistochemistry Manual Detection Kit (Akoya Biosciences), according to the manufacturer’s protocol. The antibodies for mIF were listed in Extended Data Table 6. Briefly, after deparaffinization, rehydration, and blocking endogenous peroxidase, microwave treatment (MWT) was used in the Opal method to quench endogenous peroxidase activity, for antigen retrieval (AR), and to remove antibodies after a target has been detected. MWT was first performed at 100% power until the boiling point is reached and then 20% power for 15 minutes in AR6 or AR9 solutions provided by the kit. The sections were cooled down at room temperature for 15 minutes and washed in 1X Tris Buffered Saline with Tween 20 (TBST) and blocked with blocking/antibody diluent for 10 minutes, before being incubated with primary antibody for 60 minutes. Sections were incubated with polymer anti-mouse or rabbit horseradish peroxidase (HRP) for 10 min, followed by incubation with an Opal fluorophore (Opal480, Opal520, Opal570, Opal620, Opal690, or Opal780) for 10 minutes. Bound primary and secondary antibodies were then eluted with MWT treatment. After washing in H_2_O and 1X TBST, the process of staining and antibody removal was repeated using a different Opal fluorophore. The sequence of antibodies, AR, and fluorophore used in this study are listed in Table X. Finally, after staining with the sixth Opal fluorophore, tissue specimens were stained with 4’,6-diamidino-2-phenylindole (DAPI) for 5 minutes and mounted in ProLong Diamond Antifade Mountant (ThermoFisher Scientific).

Vectra Polaris Automated Quantitative Pathology Imaging System (Akoya Biosciences) was used for multispectral imaging at 20x magnification. Whole slide images were loaded into QuPath for quantification. QuPath, custom R and python scripts were used for image analysis to determine the xy coordinates of cells within tissues slides, measure fluorescence intensity within each cell, calculate cellular density, and create spatial maps of features within the tissue.

### Statistical analysis

The objective response rate (ORR) is reported as 35% in week 12 after the administration of cabozantinib. Simon’s minimax two-stage design was adopted for a possible early termination for futility. In the approved study protocol, we hypothesized that there is >24% response rate at 12-week and a rate <5% was considered futility. In the first stage, 11 patients were accrued (this does not include screen failures), and if there are no responses among them, the study was stopped for futility. Otherwise, an additional 6 patients were accrued for a total of 17 patients. The null hypothesis was rejected if there were 3 or more responses in 17 patients. The design yields a type I error rate of 0.05 and power of 80% when the true response rate is 24%. The final response rate was estimated with 95% confidence interval by binomial test.

The OS and DFS were estimated with the Kaplan-Meier method along with 95% CI. For the biomarker study, descriptive statistics were used to summarize biomarker endpoints. Depending on whether data is normally distributed, unpaired t-test, Mann-Whitney or Wilcoxon rank sum tests were used to compare each biomarker between any two groups stratified by response or other factors. For more details, please refer to Supplementary Tables 1 – 12.

FACS and IF data are shown from a representative experiment. Statistical analysis was done using GraphPad Prism (v9) software or R package. All statistical tests were described in figure legends.

## Extended Data

**Extended Data Table 1|:** Baseline characteristics

		N	%

	Total	17	100

Age at Time of Study	Median (Range)	58 (42–86)	

Gender	Male	14	82.4
	Female	3	17.6

Race	White	14	82.4
	Black	2	11.8
	Hispanic/Other	1	5.9

Clinical TNM Stage	T3N0M0	15	88.2
	T4N0M0	2	11.8

Eastern Cooperative Oncology Group Performance Status	0	9	52.9
	1	8	47.1

Median Baseline Tumor Size (Range, cm)	9.62 (3.31 – 24.41)		

**Extended Data Table 2|:** Summary of adverse events

Event		Any Grade		≥ Grade 3	
Treatment - Related AEs		N	%	N	%
	Diarrhea	12	70.6	0	0
	Anorexia	10	58.8	1	5.9
	Fatigue	10	58.8	2	11.8
	Hypertension	10	58.8	4	23.5
	Nausea	9	52.9	0	0
	Palmar-plantar erythrodysesthesia syndrome	9	52.9	5	29.4
	Mouth sores	8	47.1	2	11.8
	Alanine aminotransferase increased	6	35.3	0	0
	Hypomagnesemia	4	23.5	0	0
Treatment - Related SAEs	Pulmonary embolism	1	5.9		
Dose Reductions Due to Treatment - Related AEs	40 mg	5	29.4		
	20 mg	2	11.8		
Post-operative Surgical Complications	Acute blood loss anemia	2	11.8	0	0
	Ileus	1	5.9	0	0
	Clostridioides difficile infection	1	5.9	0	0
	Urinary retention	1	5.9	0	0
	Urine leak	1	5.9	0	0
	Acute kidney injury	1	5.9	0	0
	Wound infection	1	5.9	0	0
	Evisceration	0	0	1	5.9
	Pulmonary embolism	1	5.9	0	0

**Extended Data Table 3|:** Summary of clinical and pathological data

Patient #	Clinical Stage	Best Response	Tumor Size (Cm)	Pathologic Stage (ypT)	WHO/ISUP Grade	Therapy related changes	Necrosis
1	T3	PR	8.7	pT3a	2	(70%) Intra/peritumoral hyalinization, hemorrhage, calcifications, cholesterol clefts, foreign body-type giant cells, chronic inflammation, perivascular fibrosis/hyalinization	Yes
2	T3	PR	4.9	pT3b	4	(60%) Intra/peritumoral hyalinization, cystic changes, hemorrhage, chronic inflammation, perivascular fibrosis/hyalinization	Yes
3	T3	SD	N/A	N/A	N/A	Biopsy: Renal cell carcinoma with eosinophilic and clear cell features	No
4	T3	SD	6.1	pT3a	4 (Rhabdoid and sarcomatoid)	(60%) Intra/peritumoral hyalinization, cystic changes, hemorrhage, chronic inflammation, perivascular fibrosis/hyalinization	Yes
5	T3	PR	11	pT3a	4 (Sarcomatoid)	(70%) Intra/peritumoral hyalinization, hemorrhage, calcifications, cholesterol clefts, foreign body-type giant cells, chronic inflammation, perivascular fibrosis/hyalinization	Yes
6	T4	PR	10	pT3a	3	(70%) Intra/peritumoral hyalinization, cystic changes, hemorrhage, chronic inflammation, perivascular fibrosis/hyalinization	Yes
7	T3	SD	2	pT1a	2	(90%) Intra/peritumoral hyalinization, cystic changes, hemorrhage, chronic inflammation, perivascular fibrosis/hyalinization	No
8	T3	SD	7.7	pT3a	3	(60%) Intra/peritumoral hyalinization, hemorrhage, calcifications, chronic inflammation, perivascular fibrosis/hyalinization	Yes
9	T3	SD	5.7	pT1b	3	(30%) Intra/peritumoral hyalinization, hemorrhage, cholesterol clefts, foreign body-type giant cells, chronic inflammation, perivascular fibrosis/hyalinization	Yes
10	T4	SD	12.5	pT4	4 (Rhabdoid and sarcomatoid)	(10%) Intra/peritumoral hyalinization, hemorrhage, chronic inflammation, perivascular fibrosis/hyalinization	Yes
11	T3	SD	9.5	pT3a	2	(70%) Intra/peritumoral hyalinization, cystic changes, hemorrhage, perivascular fibrosis/hyalinization	No
12	T3	SD	14.5	pT3a	4 (Sarcomatoid)	(30%) Intra/peritumoral hyalinization, hemorrhage, cholesterol clefts, foreign body-type giant cells, chronic inflammation, perivascular fibrosis/hyalinization	Yes
13	T3	PR	3	pT3a	3	(15%) Intra/peritumoral hyalinization, hemorrhage, chronic inflammation, perivascular fibrosis/hyalinization	No
14	T3	PR	5.2	pT3a	3	(30%) Intra/peritumoral hyalinization, cystic changes, hemorrhage, chronic inflammation, perivascular fibrosis/hyalinization	No
15	T3	SD	9.8	pT3c	3	(60%) Intra/peritumoral hyalinization, hemorrhage, calcifications, cystic changes, cholesterol clefts, foreign body-type giant cells, chronic inflammation, perivascular fibrosis/hyalinization	Yes
16	T3	SD	8.7	pT3a	2	(30%) Intra/peritumoral hyalinization, hemorrhage, cystic changes, cholesterol clefts, foreign body-type giant cells, chronic inflammation, perivascular fibrosis/hyalinization	Yes
17	T3	SD	10.5	pT3a	3	(20%) Intra/peritumoral hyalinization, hemorrhage, cystic changes, cholesterol clefts, foreign body-type giant cells, chronic inflammation, perivascular fibrosis/hyalinization	Yes

**Extended Data Table 4|:** Somatic variants detected in ccRCC patients received cabozantinib

Patient	Timepoint	Gene	Mutation nucleotide	Mutation amino acid	VAF %
1	Baseline	SETD2	CTAAG>C	Y501fs	0.067
3	W6D1	TP53	TGAG>C	L252del	2.35
	W6D1	TP53	C>T	M237I	0.47
4	W6D1	NRAS	C>T	G12D	0.07
4	Post Tx	NRAS	C>T	G12D	0.18
5	Baseline	VHL	TC>T	R167fs	0.58
10	W6D1	VHL	GTC>G	P81fs	0.3
12	W6D1	VHL	ACCCAAATGTG>A	P192fs	0.09
22	W6D1	TERT	T>G	*UTR:* c.−57A>C	0.13

**Extended Data Table 5|:** Flow cytometry antibodies

Target	Fluorochrome	Clone	Source
CD1c	BUV395	F10/21 A3	BD Biosciences
CD16	BUV496	3G8	BD Biosciences
CD14	BUV661	M5E2	BD Biosciences
CD11b	BUV737	ICRF44	BD Biosciences
CD56	BV421	HCD56	BioLegend
CD8a	BV605	RPA-T8	BioLegend
CD38	BV650	HB-7	BioLegend
CD11c	BV711	3.9	BioLegend
CD20	BV785	2H7	BioLegend
CD141	FITC	AD5–14H12	Miltenyi Biotec
CD71	PerCP/Cy5.5	CY1G4	BioLegend
CD66b	PE	6/40c	BioLegend
CD4	PE/Dazzle 594	OKT4	BioLegend
IgD	PE/Cy7	IA6–2	BioLegend
CD19	APC	HIB19	BioLegend
CD3	A700	UCHT1	BioLegend
HLA-DR	APC/Cy7	I243	BioLegend
CD25	BUV395	M-A251	BD Biosciences
CD4	BUV496	OKT4	BD Biosciences
PD1	BUV737	EH12.1	BD Biosciences
CD39	BV421	A1	BioLegend
CD38	BV650	HB-7	BioLegend
Ki67	BV711	B56	BD Biosciences
CD45RA	BV785	HI100	BioLegend
TCF1/TCF7	A488	C63D9	Cell Signaling Technology
CD3	PerCP	UCHT1	BioLegend
TIM-3	PE		R&D Systems
FOXP3	PE-eFluor 610	PCH101	Invitrogen
CD28	PE-Cy7	CD28.2	Invitrogen
CCR7	APC	G043H7	BioLegend
Granzyme B	A700	GB11	BD Biosciences
CD16	BUV661	3G8	BD Biosciences
Ki67	BV650	B56	BD Biosciences
CD141	PE	AD5–14H12	Miltenyi Biotec
CD11b	APC	ICRF44	BD Biosciences
Fixable Live Dead	Aqua		Thermo-Fisher

**Extended Data Table 6|:** Multiplex immunofluorescence antibodies

Target	Clone	Source	Concentration	Antigen Retrieval	Fluorophore
CD8	C8/144B	Invitrogen	1:500	AR9	690
FoxP3	236A/E7	Abcam	1:100	AR9	620
TCF1	C63D9	Cell Signaling Technology	1:100	AR6	520
αSMA	1A4	Invitrogen	1:150	AR6	570
MHC-II	Tu39	BioLegend	1:50	AR6	480
CD4	EPR6855	Abcam	1:150	AR9	780

## Data Availability

RNA-Seq data has been deposited to the NCBI Gene Expression Omnibus (GEO) database.
